# Community-based interventions to prevent serious complications following spinal cord injury in Bangladesh: the CIVIC trial statistical analysis plan

**DOI:** 10.1186/s13063-019-3181-2

**Published:** 2019-04-25

**Authors:** Robert D. Herbert, Lisa A. Harvey, Mohammad S. Hossain, Md. Shofiqul Islam, Qiang Li, Laurent Billot, The CIVIC Trial Collaboration, Md. Akhlasur Rahman, Stephen Muldoon, Stephen Jan, Valerie Taylor, Ian D. Cameron, Harvinder Singh Chhabra, Richard I. Lindley, Fin Biering-Sørensen

**Affiliations:** 1 Neuroscience Research Australia (NeuRA) and University of New South Wales, Sydney, Australia; 2 John Walsh Centre for Rehabilitation Research, Sydney Medical School, The University of Sydney, Sydney, Australia; 3 The Centre for the Rehabilitation of the Paralysed, Savar, Bangladesh; 4 The George Institute for Global Health and Faculty of Medicine, University of New South Wales, Sydney, Australia

**Keywords:** Spinal cord injury, Community-based rehabilitation, Clinical trial, Secondary complications

## Abstract

**Background:**

People who sustain spinal cord injuries in low- and middle-income countries are vulnerable to life-threatening complications after discharge. The aim of this trial is to determine the effect on all-cause mortality of a sustainable model of community-based care provided over the first 2 years after discharge.

**Methods and analysis:**

The CIVIC trial is a single centre, parallel group trial with concealed and stratified randomisation. The protocol has been previously published (*BMJ Open* 2016;6:e010350). This paper provides the accompanying detailed statistical plan. In total, 410 people with recent spinal cord injury who are wheelchair dependent and about to be discharged from the Centre for the Rehabilitation of the Paralysed in Bangladesh are randomised to intervention or control groups. Participants assigned to the intervention group receive a model of community-based care in which a case manager provides ongoing telephone-based support and visits participants in their homes over a 2-year period. Participants assigned to the control group receive usual care which may involve a follow-up phone call or a home visit. The primary outcome is all-cause mortality at 2 years as determined by a blinded assessor (Bangladesh does not have a death registry). The primary effectiveness analysis will compare Kaplan-Meier survival curves (time from allocation to death) in the intervention and control groups using the log-rank test (two-tailed α = 0.05). Participants will be censored at the time they were last known to be alive or at the time of the follow-up assessment. Recruitment finished in March 2018 and the last assessment will be conducted in March 2020.

**Discussion:**

The CIVIC trial will provide unbiased and precise estimates of the effectiveness of a model of community-based care for people with spinal cord injuries in Bangladesh. The results will have implications for provision of health services for people with spinal cord injuries and other conditions that cause serious disability in low-income and middle-income countries.

**Trial registration:**

ANZCTR, ACTRN12615000630516, U1111-1171-1876. Registered on 17 June 2015.

**Electronic supplementary material:**

The online version of this article (10.1186/s13063-019-3181-2) contains supplementary material, which is available to authorized users.

## Background

While it is apparent that spinal cord injuries are common in low- and middle-income countries, robust incidence data are scarce [1–4] and robust population-based data on mortality rates following spinal cord injuries in low- and middle-income countries are not available. A longitudinal cohort study of a representative sample of 350 people with spinal cord injury who survived until discharge from a specialised hospital in Bangladesh found that one in five people who were wheelchair-dependent at discharge had died within 2 years [5]. Most died from complications related to pressure ulcers. The problems of pressure ulcers in low- and middle-income countries is widely documented [1, 6].

Our research team, which includes health professionals and health service providers based in Bangladesh, has proposed an inexpensive model of community-based care for people discharged from hospital with spinal cord injury. The objective is to increase survival after discharge. The model of care involves assigning a case manager to each person with spinal cord injury at the time the person is discharged from hospital. The case manager telephones the person each fortnight in the first year following discharge and each month in the second year, and visits the person in their home three times over the first 2 years. At each point of contact, the case manager screens for complications and provides the person and their families with ongoing advice, support, and education. There is a particular focus on preventing and treating pressure ulcers.

The trial commenced in July 2015 and the last participant was randomised in March 2018. The trial is due for completion in March 2020. The protocol for the CIVIC trial has been published [7]. The purpose of this paper is to provide the detailed statistical analysis plan and allow future readers of the trial report to confirm that the trial has been analysed according to a pre-specified plan. The study will include a formal cost-effectiveness analysis and a process analysis [8], but they are not described in this statistical analysis plan.

## Methods/design

### Aim

The primary aim of the CIVIC trial is to determine whether a sustainable community-based model of care reduces all-cause mortality 2 years after discharge in people with spinal cord injury in Bangladesh. Secondary aims are to determine whether this model of care reduces the burden of complications, reduces the prevalence and severity of pressure ulcers, reduces depression, enhances quality of life, independence, and participation, and is cost-effective.

### Design

The trial is a two-arm parallel pragmatic randomised trial. It is investigator-driven. The trial is managed by George Clinical, India.

### Setting

The trial is being conducted at the Centre for the Rehabilitation of the Paralysed in Savar, Bangladesh. This is a not-for-profit hospital that provides care and rehabilitation for people with spinal cord injuries. It admits approximately 350 people with recent spinal cord injuries each year making it one of the largest spinal injury units catering for recently injured people with neurological loss in Asia and the only specialised centre for spinal cord injuries in Bangladesh.

### Participants

In total, 410 people have been randomised to the CIVIC trial. Participants are people who, at the time of randomisation, had been admitted to the Centre for Rehabilitation of The Paralysed with an acute spinal cord injury, and who were over the age of 16 years and were wheelchair-dependent. Potential participants were excluded if walking was their usual mode of ambulation or they planned to move to another country.

### Procedures

The full protocol can be found elsewhere [7]. In brief, participants were randomised in a 1:1 ratio to an intervention or control group using randomly permuted blocks. The allocation sequence was stratified by level of lesion (paraplegia or tetraplegia) using the user-written ralloc command in Stata [9]. Participants in the intervention group receive fortnightly phone calls from a case manager in the first year after discharge and monthly phone calls in the second year. They also receive three home visits over the 2 years and up to AUD $80 to spend on necessary items. Participants in the control group receive standard care only. Standard care may consist of a phone call or a home visit.

The primary outcome is survival (all-cause mortality) at 2 years after randomisation determined by a blinded assessor. Bangladesh does not have a death registry, and so the date of death is confirmed by interviewing next of kin or carers at 2 years. Wherever possible, independent corroboration of the date of death is obtained. There are a number of secondary outcomes, including burden of complications, prevalence and severity of pressure ulcers, depression, quality of life, independence, and participation. Questionnaires are administered in the Bangla language under the guidance of a blinded assessor.

### Data management and data integrity

Data are collected in paper format, transferred to George Clinical India, and entered into an electronic database (RedCap). Electronically transcribed data are stored and managed by the Data Management Division of George Clinical India. Data are double-entered. Automated checks are conducted to detect data entry errors. Data queries are emailed to the site coordinator and stored on the database.

### Sample size

The sample size of 410 gives a better than 80% probability of detecting an increase in survival from 83% to 93% at 2 years with a two-sided log-rank test, uniform follow-up time of 2 years, loss to follow-up in both groups of 15% at 2 years, and α of 0.05.

Allowance has been made in sample size calculations for a single interim analysis conducted when the first 205 participants have been followed up (i.e. at an information fraction of 205/410 = 0.5) using the O’Brien-Fleming alpha spending function.^1^

### Stopping rules

A recommendation to terminate the trial early for effectiveness will only be made if the Data Monitoring Committee determines both that there is proof beyond reasonable doubt that the intervention is clearly indicated (that is, the net benefit—weighing the health benefits against costs, risks, and inconveniences—clearly favours intervention) and that the trial provides sufficiently strong evidence of benefit that it might reasonably be expected to influence patient care. A recommendation to terminate the trial early for safety will only be made if the Data Monitoring Committee determines there is proof beyond reasonable doubt that the intervention causes an unacceptable net harm. The trial will not be terminated on the grounds of futility.

A recommendation to terminate the trial will be informed both by a formal interim statistical analysis and other considerations, including the pattern of effects across all effectiveness and safety outcomes. The statistical criterion for termination of the trial is that the confidence interval for a beneficial effect includes only clinically important beneficial effects, or that the confidence interval includes only clinically important harmful effects. A statistically significant test of the null hypothesis of no effect will not, on its own, be grounds for termination of the trial. A formal interim analysis will be conducted by an independent statistician and presented to the Data Monitoring Committee after outcomes have been obtained from approximately 205 participants. The Steering Committee will not be informed of the results of the unblinded interim analysis unless a recommendation is made to terminate the trial.

### Statistical analysis

The analysis will be conducted by statisticians from the George Institute using SAS. Efficacy analyses will be independently replicated by one of the investigators using Stata. Any discrepancies between the two analyses will be resolved by consensus.

### General principles

Analyses will be conducted on an intention-to-treat basis. Hypothesis tests will be conducted but the interpretation of the trial findings will consider point estimates of effects and their confidence intervals. Hypothesis tests will be two-tailed tests (alpha = 0.05). Confidence intervals and *p* values will not be adjusted for multiplicity, but interpretation of secondary outcomes will include consideration of multiplicity.

### Trial profile

The flow of participants through the study will be reported in a CONSORT flow diagram. Reasons for exclusion will be provided.

### Description of study sample at baseline

The study sample will be described in detail using data obtained prior to randomisation. Formal between-group comparisons will not be made on baseline variables.

### Adherence

Data will be obtained on adherence to the trial protocol. For the primary trial report, adherence will be reported as the number and duration of calls received or the number of home visits made and these will be expressed as a proportion of the number of calls or home visits specified in the protocol. The denominator of this proportion will take into account that calls and home visits cannot occur after a participant has died.

### Efficacy analysis: primary outcome

#### Primary analysis

The primary effectiveness analysis will compare time to death from any cause in the intervention and control groups. Kaplan-Meier survival curves will be compared using the log-rank test (two-tailed α = 0.05). Participants will be censored at the time they were last known to be alive or at the time of the follow-up assessment (intended to be 2 years after randomisation), whichever is earlier.

#### Size of effect

The primary estimates of the size of the effect of the intervention will not be adjusted for covariates. Effect estimates will be expressed as:

- a hazard ratio calculated from a simple Cox model (containing a term for intervention) with 95% confidence limits.
- both the difference and ratio of the restricted mean survival times of the intervention and control groups at 2 years, with 95% confidence limits. Restricted mean survival times will be estimated by numerical integration of the Kaplan-Meier curves up to 2 years. Confidence intervals will be generated using the procedures described by Cronin and colleagues [10].
- the difference in risk of all-cause mortality at 2 years, with 95% confidence limits. The confidence interval will be bounded by Wald (asymptotic) confidence limits based on the normal approximation.

If there is any discrepancy between the log rank test used in the primary analysis and the test of the size of effect implicit in the confidence intervals for estimates of the size of the effect, the log rank test will be used as the primary test of effect.

#### Sensitivity analyses

Additional tests will be conducted using:

- a Cox model adjusted for level of lesion (tetraplegia or paraplegia).
- a combined test of restricted mean survival times adjusted for level of lesion (tetraplegia or paraplegia) [11].
- a test of the difference in all-cause mortality at 2 years adjusted for level of lesion (tetraplegia or paraplegia) using log-binomial regression.

#### Missing data handling

For the analysis of time to death missing data will not be imputed. Instead, participants with an unknown vital status at 2 years will be censored when they were last known to be alive. For the comparison of all-cause mortality at 2 years, if more than 5% of participants have an unknown vital status, a further sensitivity analysis will examine the treatment effect under all possible outcomes (dead or alive) for all participants with a missing data endpoint [12]. Within each treatment arm, if we denote as m_k_ (k = 0,1) the number of participants with a missing outcome, we will run m_k_ + 1 possible scenarios from the most to the least favourable where:

- Scenario 0: 0 participants died
- Scenario 1: 1 participant died
- Scenario 2: 2 participants died
- …
- Scenario m_k_: m_k_ participants died

For each of the resulting (m_0_ + 1) × (m_1_ + 1) combinations, we will calculate a contingency table and associated chi-square *p* value and examine which combinations are consistent with the non-imputed analysis. This will tell us how extreme the missing data assumption would need to be to provide a result that is different to the non-imputed analysis.

#### Subgroup analyses

A subgroup analysis will examine whether the effect of intervention is moderated by level of lesion (paraplegia or tetraplegia) or age (< 30, 30–50, > 50 years). The subgroup analysis will be conducted on the time to death outcome using a Cox model with terms for intervention, level of lesion (or age), and the intervention by level of lesion (or age) interaction.

### Efficacy analysis: secondary outcomes

Between-group comparisons of secondary outcomes will be conducted using linear models, adjusting only for the stratification and baseline variables. In these models, the outcome will be a linear function of intervention and level of lesion (tetraplegia or paraplegia). For continuous outcomes, baseline scores will be included in the model to increase statistical precision and statistical power. The effect of intervention on continuous outcomes will be estimated as the adjusted mean difference and 95% confidence interval. For binary outcomes, log-binomial regression will be used. The effect of intervention on binary outcomes will be estimated as the adjusted ratio of proportions and 95% confidence interval.

#### Missing data handling

The efficacy analysis of secondary endpoints will use all available data. Missing data will not be imputed.

#### Complier average effects and survivor average effects

If there is substantial non-compliance with the intervention (fewer than 75% of planned phone contacts or home visits), the complier average causal effect of intervention on all-cause mortality at 2 years will be estimated. The number of phone contacts and the number of home visits with participants in the intervention group will be used to quantify adherence to the protocol by participants in the intervention group. It will be assumed that participants in the control group are unable to access the intervention. The complier average causal effects will be estimated using instrumental variable regression [13].

If there is a substantially different survival in the intervention and control groups (greater than 5% absolute difference in survival at 2 years), a sensitivity analysis will be conducted to determine the plausible range of survivor average causal effects on secondary outcomes using the method described by Chiba and Vanderweele [14].

### Safety analysis

The safety analysis will consist of documentation of serious adverse events, deaths, hospitalisations, and events resulting in persistent or significant disability. Comprehensive safety data will be obtained from participants in the intervention group over the course of the trial because the research team will be in regular contact with participants in the intervention group. In contrast, incomplete safety data will be obtained from participants in the control group over the course of the trial because the research team has little or no contact with participants in the control group until follow-up at 2 years. The closer monitoring of intervention group participants over the course of the trial generates a potential ascertainment bias which makes interpretation of these safety data potentially misleading. For that reason, we will not conduct formal between-group comparisons of safety data collected over the course of the trial and we do not anticipate providing details of this information in the primary trial report. Instead, the efficacy analyses will be used to provide insights into safety because the primary outcome and many of the secondary outcomes reflect adverse events.

### Figures and tables

**Fig. 1:**
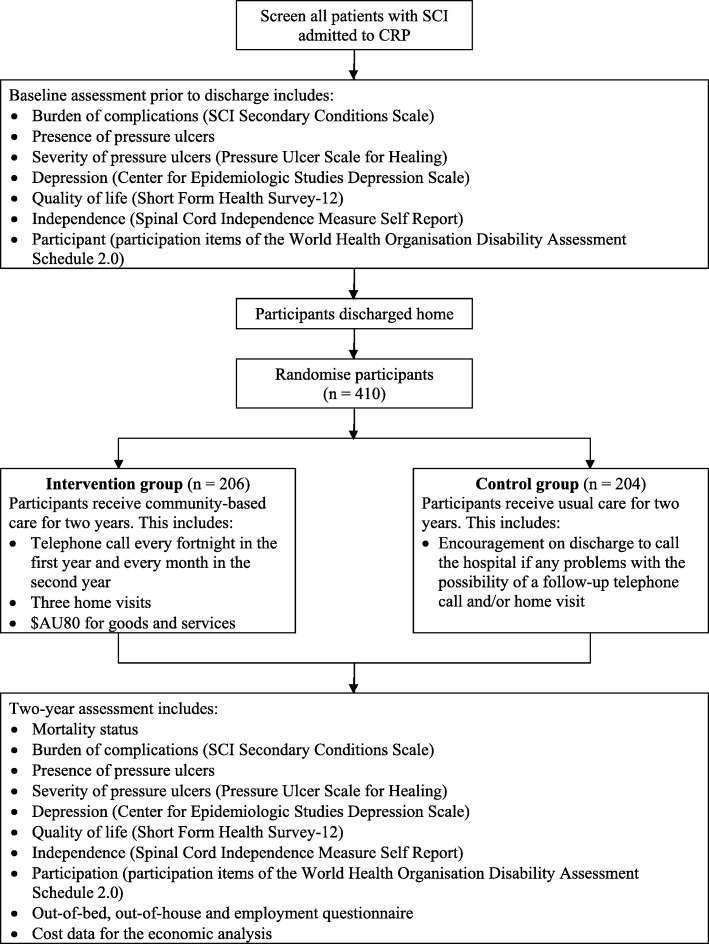
The CONSORT flow chart. CRP Centre for the Rehabilitation of the Paralysed, SCI spinal cord injury

The final report will include the CONSORT flow chart (Fig. 1) and five tables (Additional file 1).

## Discussion

This paper presents the statistical analysis plan for the CIVIC trial. By publishing the statistical analysis plan while the trial is still underway, we can subsequently demonstrate, when the trial report is produced, that the data were analysed according to a pre-specified plan. Readers of the trial report will be able to check if the trial was subject to post-hoc or data-driven analyses.

### Changes from the register and published study protocols

This detailed statistical plan includes two minor changes to the statistical analysis procedures described briefly in the trial register and the published protocol. They are:

1. The register indicates that between-group comparisons of binary secondary outcomes will be conducted using logistic regression, but instead log-binomial regression will be used.
2. The published protocol indicates that multiple imputation will be used if more than 5% of data are missing for a particular analysis. Instead, efficacy analyses will be conducted on all available data without imputation. An analysis of the sensitivity of findings to missing data will be conducted using the all-cause mortality outcome.

This statistical analysis plan supersedes the information previously provided in the registry and the published protocol. The registry and the working version of the protocol will be updated to make them consistent with this statistical analysis plan.

## Trial status

Key dates in the conduct of the trial are as follows:

- The trial was registered on 17 June 2015 with the Australian and New Zealand Clinical Trial Registry (https://www.anzctr.org.au/Trial/Registration/TrialReview.aspx?id=368756).
- The first participant was randomised on 12 July 2015.
- The last participant was randomised on 19 March 2018.
- The first participant finished the trial on 3 August 2017.
- The last participant will finish the trial in March 2020.
- The trial protocol was submitted for publication on 23 October 2015 [7].
- This statistical plan is Version 5, dated 12 April 2018.
- This statistical plan was ratified on 12 April 2018 prior to inspection of the data.

## Additional file

Additional file 1: Shells for the five tables that will be included in the final report of the trial (do not include data). (DOCX 45 kb)

## Footnotes

1 In Stata: landemets, alpha(0.05) method(obf) t(0.5(0.5)1) matrix LANDEMETS = r(bound_alpha) local OBFalpha = LANDEMETS[2, 5] stpower logrank 0.834 0.934, power(0.8) alpha(`OBFalpha’) wdprob(0.15) (Note: the landemets command is a user-written command.)
