# Supplementary material for: Community-based interventions to prevent serious complications following spinal cord injury in Bangladesh: the CIVIC trial statistical analysis plan
Source: Trials. 2019 Apr 25;20:238. doi: 10.1186/s13063-019-3181-2 (PMC6485052; doi:10.1186/s13063-019-3181-2)
Supplement: Supplementary file 1 — Shells for the five tables that will be included in the final report of the trial (do not include data). (DOCX 45 kb) [file 13063_2019_3181_MOESM1_ESM.docx]

**Additional file**: Shells for the 5 Tables that will be included in the final report of the trial.

**Table 1.** Characteristics of participants at baseline.

|  | **Control**  **(n = XXX)** | **Intervention**  **(n = XXX)** | **All participants**  **(n = XXX)** |
| --- | --- | --- | --- |
| **Age (years)^1^** | n=XXX  XX.X (XX.X to XX.X) | n=XXX  XX.X (XX.X to XX.X) | n=XXX  XX.X (XX.X to XX.X) |
| **Time since injury (months)^1^** | n=XXX  XX.X (XX.X to XX.X) | n=XXX  XX.X (XX.X to XX.X) | n=XXX  XX.X (XX.X to XX.X) |
| **Male : female^2^** | n=XXX  XXX (XX.X%) : YYY (YY.Y%) | n=XXX  XXX (XX.X%) : YYY (YY.Y%) | n=XXX  XXX (XX.X%) : YYY (YY.Y%) |
| **Neurological level^2^**  **C1 to C4**  **C5 to C8**  **T1 to T7**  **T8 to T12**  **L1 to L5**  **S1 to S5** | n=XXX  XX (XX.X%)  XX (XX.X%)  XX (XX.X%)  XX (XX.X%)  XX (XX.X%)  XX (XX.X%) | n=XXX  XX (XX.X%)  XX (XX.X%)  XX (XX.X%)  XX (XX.X%)  XX (XX.X%)  XX (XX.X%) | n=XXX  XX (XX.X%)  XX (XX.X%)  XX (XX.X%)  XX (XX.X%)  XX (XX.X%)  XX (XX.X%) |
| **ASIA Impairment Scale^2^**  **A**  **B**  **C**  **D** | n=XXX  XX (XX.X%)  XX (XX.X%)  XX (XX.X%)  XX (XX.X%) | n=XXX  XX (XX.X%)  XX (XX.X%)  XX (XX.X%)  XX (XX.X%) | n=XXX  XX (XX.X%)  XX (XX.X%)  XX (XX.X%)  XX (XX.X%) |
| **Total motor score (/100)^1^** | n=XXX  XX (XX to XX) | n=XXX  XX (XX to XX) | n=XXX  XX (XX to XX) |
| **Marital status^2^**  **Married**  **Never married**  **Separated/divorced**  **Widowed** | n=XXX  XX (XX.X%)  XX (XX.X%)  XX (XX.X%)  XX (XX.X%) | n=XXX  XX (XX.X%)  XX (XX.X%)  XX (XX.X%)  XX (XX.X%) | n=XXX  XX (XX.X%)  XX (XX.X%)  XX (XX.X%)  XX (XX.X%) |
| **In paid employment prior to injury (yes/no)^2^** | n=XXX XXX (XX.X%) : YYY (YY.Y%) | n=XXX  XXX (XX.X%) : YYY (YY.Y%) | n=XXX  XXX (XX.X%) : YYY (YY.Y%) |
| **Monthly income prior to injury (BDT)^1, 3^** | n=XXX  XX (XX to XX) | n=XXX  XX (XX to XX) | n=XXX  XX (XX to XX) |
| **Primary carer^2^**  **Spouse**  **Parent**  **Child**  **Other** | n=XXX  XX (XX.X%)  XX (XX.X%)  XX (XX.X%)  XX (XX.X%) | n=XXX  XX (XX.X%)  XX (XX.X%)  XX (XX.X%)  XX (XX.X%) | n=XXX  XX (XX.X%)  XX (XX.X%)  XX (XX.X%)  XX (XX.X%) |

**Legend:**

^1^ Medians (first and third quartiles). ^2^ Counts (column percentages).
^3^ 1 Bangladeshi Taka ≈ 0.0128 USD, 0.0113 EUR or 0.0098 GBP.

*Abbreviations*:

ASIA: American Spinal Injuries Association**Table 2. Details of the Intervention.**

| **Number of telephone contacts^1^** | n = XXX  XX (XX to XX) |
| --- | --- |
| **Proportion of target number of telephone contacts^2^** | n = XXX  XX (XX to XX) |
| **Duration of telephone contacts (minutes)^1^** | n = XXX  XX (XX to XX) |
| **Number of home visits^1^** | n = XXX  XX (XX to XX) |
| **Proportion of target number of home visits^2^** | n = XXX  XX (XX to XX) |

**Legend:**

^1^ Medians (first and third quartiles). ^2^ Numerator and denominator do not include telephone contacts or home visits after participant died.

**Table 3.** Effect of the intervention on survival at two years.

|  | **Method** | **Estimate (95% CI)** | **p** |
| --- | --- | --- | --- |
| **Hazard ratio** | Cox model, no covariates | X.XX (X.XX to X.XX) | 0.XXX |
| **Hazard ratio** | Cox model, adjusted for level of lesion | X.XX (X.XX to X.XX) | 0.XXX |
| **Difference in RSMT (months)** | Method of Cronin et al | X.X (X.X to X.X) | 0.XXX |
| **Difference in RSMT (months)** | Method of Cronin et al, adjusted for level of lesion | X.X (X.X to X.X) | 0.XXX |
| **Ratio of RSMTs** | Method of Cronin et al | X.XX (X.XX to X.XX) | 0.XXX |
| **Ratio of RSMTs** | Method of Cronin et al, adjusted for level of lesion | X.XX (X.XX to X.XX) | 0.XXX |
| **Risk difference** | Wald CIs | 0.XX (0.XX to 0.XX) | 0.XXX |
| **Risk difference** | Wald CIs, adjusted for level of lesion | X.XX (X.XX to X.XX) | 0.XXX |

**Legend:**

*Abbreviations*: RMST: restricted mean survival time.

**Table 4.** Effect of the intervention on the continuous secondary outcomes.

|  | **Baseline** | | **Follow-up (two years)** | | | | | | |  |
| --- | --- | --- | --- | --- | --- | --- | --- | --- | --- | --- |
|  |  |  | **Unadjusted** | | **Adjusted for baseline value and  level of lesion (paraplegia or tetraplegia)** | | | | | |
|  | **Control^1^**  **(n = XXX)** | **Inter-vention^1^**  **(n = XXX)** | **Control^1^**  **(n = XXX)** | **Inter-vention^1^**  **(n = XXX)** | | **Control^2^**  **(n = XXX)** | **Inter-vention^2^**  **(n = XXX)** | **Differ-ence^3^**  **(n = XXX)** | **p** | |
| **SCI-SCS (/49)** | XX.X (XX.X)  XX.X(XX.X, XX.X) | XX.X (XX.X)  XX.X(XX.X, XX.X) | XX.X (XX.X)  XX.X(XX.X, XX.X) | XX.X (XX.X)  XX.X(XX.X, XX.X) | | XX.X (XX.X) | XX.X (XX.X) | XX  (XX.X, XX.X) | 0.XXX | |
| **PUSH (/17)** | XX.X (XX.X)  XX.X(XX.X, XX.X) | XX.X (XX.X)  XX.X(XX.X, XX.X) | XX.X (XX.X)  XX.X(XX.X, XX.X) | XX.X (XX.X)  XX.X(XX.X, XX.X) | | XX.X (XX.X) | XX.X (XX.X) | XX  (XX.X, XX.X) | 0.XXX | |
| **CESD-R (/60)** | XX.X (XX.X)  XX.X(XX.X, XX.X) | XX.X (XX.X)  XX.X(XX.X, XX.X) | XX.X (XX.X)  XX.X(XX.X, XX.X) | XX.X (XX.X)  XX.X(XX.X, XX.X) | | XX.X (XX.X) | XX.X (XX.X) | XX  (XX.X, XX.X) | 0.XXX | |
| **SF12 – physical (standardised)** | XX.X (XX.X)  XX.X(XX.X, XX.X) | XX.X (XX.X)  XX.X(XX.X, XX.X) | XX.X (XX.X)  XX.X(XX.X, XX.X) | XX.X (XX.X)  XX.X(XX.X, XX.X) | | XX.X (XX.X) | XX.X (XX.X) | XX  (XX.X, XX.X) | 0.XXX | |
| **SF12 – mental (standardised)** | XX.X (XX.X)  XX.X(XX.X, XX.X) | XX.X (XX.X)  XX.X(XX.X, XX.X) | XX.X (XX.X)  XX.X(XX.X, XX.X) | XX.X (XX.X)  XX.X(XX.X, XX.X) | | XX.X (XX.X) | XX.X (XX.X) | XX  (XX.X, XX.X) | 0.XXX | |
| **SCIM-SR (/100)** | XX.X (XX.X)  XX.X(XX.X, XX.X) | XX.X (XX.X)  XX.X(XX.X, XX.X) | XX.X (XX.X)  XX.X(XX.X, XX.X) | XX.X (XX.X)  XX.X(XX.X, XX.X) | | XX.X (XX.X) | XX.X (XX.X) | XX  (XX.X, XX.X) | 0.XXX | |
| **WHODAS 2.0 (/40)** | XX.X (XX.X)  XX.X(XX.X, XX.X) | XX.X (XX.X)  XX.X(XX.X, XX.X) | XX.X (XX.X)  XX.X(XX.X, XX.X) | XX.X (XX.X)  XX.X(XX.X, XX.X) | | XX.X (XX.X) | XX.X (XX.X) | XX  (XX.X, XX.X) | 0.XXX | |

**Legend:**

^1^ In each cell, the first row contains the mean (SD) and second row (where given). contains the median (first and third quartiles).
^2^ Adjusted mean and standard error. ^3^ Difference in means (95% confidence limits).

*Abbreviations:*

SCI-SCS: Spinal Cord Injury Secondary Conditions Scale

PUSH: Pressure Ulcer Scale for Healing

CESD-R: Center for Epidemiologic Studies Depression Scale revised version

SF12: Short Form Health Survey-12

SCIM-SR: Spinal Cord Independence Measure Self Report

WHODAS: World Health Organization Disability Assessment Schedule

**Table 5.** Effect of the intervention on the binary secondary outcomes.

|  | **Baseline** | | **Follow-up (two years)** | | |
| --- | --- | --- | --- | --- | --- |
|  | **Control^1^**  **(n = XXX)** | **Intervention**  **(n = XXX)** | **Control^1^**  **(n = XXX)** | **Intervention**  **(n = XXX)** | **Risk ratio^2^**  **(n = XXX)** |
| **Pressure ulcer** | XX (XX.X%) | XX (XX.X%) | XX (XX.X%) | XX (XX.X%) | XX%  (XX.X, XX.X) |
| **Bed-bound** | XX (XX.X%) | XX (XX.X%) | XX (XX.X%) | XX (XX.X%) | XX%  (XX.X, XX.X) |
| **House-bound** | XX (XX.X%) | XX (XX.X%) | XX (XX.X%) | XX (XX.X%) | XX%  (XX.X, XX.X) |
| **Unemployed** | XX (XX.X%) | XX (XX.X%) | XX (XX.X%) | XX (XX.X%) | XX%  (XX.X, XX.X) |

**Legend:**

^1^ Raw count (column percentage). ^2^ Ratio of adjusted risks (95% confidence limits) estimated with log-binomial model.
